# Socioeconomic status (SES) and cognitive outcomes are predicted by resting-state EEG in school-aged children

**DOI:** 10.1016/j.dcn.2024.101468

**Published:** 2024-10-29

**Authors:** Julie M. Schneider, Jeahong Kim, Sonali Poudel, Yune S. Lee, Mandy J. Maguire

**Affiliations:** a Louisiana State University, USA; b The University of Texas at Dallas, USA; c The University of Texas at Austin, USA

**Keywords:** Resting-state, EEG, Language development, SES, Machine-learning

## Abstract

Children’s socioeconomic status (SES) is related to patterns of intrinsic resting-state brain function that subserve relevant cognitive processes over the course of development. Although infant research has demonstrated the association between children’s environments, cognitive outcomes, and resting-state electroencephalography (rsEEG), it remains unknown how these aspects of their environment, tied to SES, impact neural and cognitive development throughout the school years. To address this gap, we applied a multivariate pattern analysis (MVPA) to rsEEG data to identify which neural frequencies at rest are differentially associated with unique aspects of socioeconomic status (SES; income and maternal education) and cognitive (vocabulary, working memory) outcomes among school-aged children (8–15 years). We find that the alpha frequency is associated with both income and maternal education, while lower gamma and theta fluctuations are tied to dissociable aspects of SES and cognitive outcomes. Specifically, changes in the gamma frequency are predictive of both maternal education and vocabulary outcome, while changes in the theta frequency are related to both income and working memory ability. The current findings extend our understanding of unique pathways by which SES influences cognitive and neural development in school-aged children.

## Highlights

• Socioeconomic status (SES) influences patterns of intrinsic resting-state brain function (rsEEG).
• These rsEEG differences subserve the development of relevant cognitive processes.
• MVPA revealed income and maternal education have dissociable effects on rsEEG.
• These differences in neural function are linked to working memory and vocabulary.
• SES relates to cognitive and neural development through unique pathways.

## 1 Introduction

Family socioeconomic status (SES) affects a number of cognitive, mental, and physical health outcomes. One of the most consistent areas affected by SES is language processing (Perkins, Finegood and Swain, 2013). However, language processing skills are multifaceted, supported by the successful development of vocabulary, phonological awareness, and syntax (Whitehurst, 1997, Hoff and Tian, 2005), as well as other cognitive skills (e.g., Christiansen and Chater, 2008), including working memory and cognitive control functions (Noble et al., 2007, Evans, 2006). Vocabulary and working memory in particular play central roles in language processing. Breadth and depth of vocabulary allows individuals to understand the meaning of what is being communicated, while working memory allows an individual to hold individual words from the speech stream in memory before integrating them into a cohesive message.

Recent research has indicated brain structure and function mediates the link between SES and these language processing skills. Notably, studies of task-free, or “resting-state,” brain function using electroencephalography (EEG) have suggested that various aspects of a child’s environment may impact the development of cortical networks (Uhlhaas et al., 2009; Uhlhaas et al., 2010), in turn affecting the neural systems underlying language processing (Cantiani et al., 2019, Tarullo et al., 2017). Despite growing evidence for the critical role of resting state EEG in clarifying the association between SES, vocabulary, and working memory, most findings are garnered from infant research. Furthermore, most studies focus on a single indicator of SES (using maternal education or income), or a composite score of SES, which hinders our ability to identify the unique mechanisms through which specific aspects of SES contribute to cognitive outcomes and their supporting neural networks (Darin-Mattsson et al., 2017, Long and Renbarger, 2023). Relatively little is known about how different indicators of SES uniquely relate to the neural, and subsequent language development of school-age children. This is especially problematic given language skills are the single best predictor of later academic achievement (Pace et al., 2019, Burchinal et al., 2016, Hoff, 2013). Therefore, the current study aims to identify how cortical networks at rest, as measured with neural oscillations, are sensitive to discrete aspects of a child’s environment (e.g., income and maternal education) and how fluctuations in these oscillations are indicative of vocabulary knowledge and working memory capacity in school-age children.

**Fig. 1:**
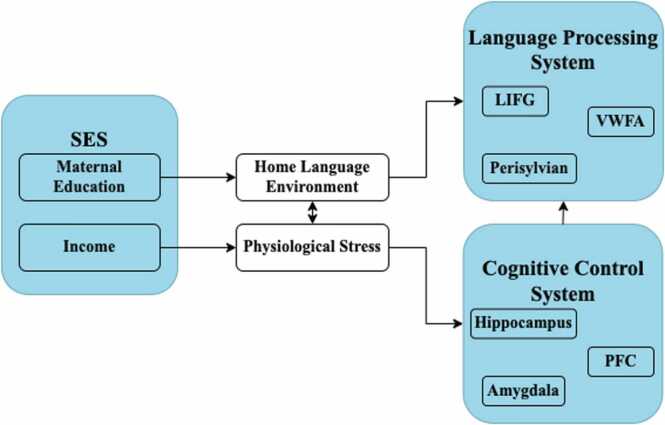
Conceptual model linking SES to language processing in the brain through the home language environment and physiological stress. This model combines those proposed by Brito and Noble (2014) and Perkins, Finegood and Swain (2013).

Several conceptual models have been proposed to account for the relationship between SES and language processing skills (Brito and Noble, 2014, Perkins et al., 2013). In general, these models are highly overlapping, agreeing that SES exerts its influence on neural development and subsequent vocabulary knowledge and working memory skills through two unique pathways (visualized in Fig. 1). The first pathway suggests that mothers with lower educational attainment provide less linguistically diverse vocabulary input to their children in the home environment, in turn affecting the development of the language processing system, including the left inferior frontal gyrus, visual word form area, and perisylvian cortex. Specifically, studies have shown that a lack of diverse vocabulary input negatively impacts child vocabulary development through language-specific neural regions, such as the left inferior frontal gyrus (Romeo et al., 2018). The second pathway suggests that a lack of income results in higher physiological stress, in turn affecting the development of the cognitive control system, including the hippocampus, prefrontal cortex, and amygdala. For example, research has shown that higher physiological stress negatively impacts cognitive control, particularly working memory mediated by the prefrontal system (Qin et al., 2009, Noble et al., 2005, Farah et al., 2006, Noble et al., 2007). As such, differences related to physiological stress may negatively (and indirectly) influence subsequent language processing abilities via this pathway of cognitive control during development.

In understanding the relationship between SES and brain development, it is necessary to establish how and why SES influences a child’s environmental experiences. SES is a complex construct that includes measures of economic resources, as well as social factors related to power, prestige and hierarchical social status (Braveman et al., 2005, Duncan and Magnuson, 2003, Krieger et al., 1997, Adler et al., 1994). Despite its complexity, most research focuses on two primary indicators: income and parental education (Hackman and Farah, 2009, Braveman et al., 2005, Duncan and Magnuson, 2003). Although often highly correlated, each of these indicators have been shown to differentially impact child development through unique pathways. In many of these studies, lower family income has a long-standing history of being linked to higher maternal physiological stress (Baum et al., 1999; Ferraro and Shippee, 2009; Ryan et al., 2021). Specifically, families’ daily lives are filled with everyday stressors stemming from household routines, finances, and tensions with partners (Helms and Demo, 2005). These daily stressors can disrupt individual and family functioning when parents are unable to adapt and prevent negative affect from compromising the quality of parent-child interactions. Stressful experiences during the day can therefore deplete parents’ self-regulation (Belsky and Jaffe, 2006; Nelson et al., 2017; Ward and Lee, 2020), impacting their ability to be patient and positively engage with their children. These daily patterns of interactions related to stressful home environments are believed to be the mechanisms through which neural differences related to income manifest.

Related to parental education, a wealth of research has established that, on average, higher levels of maternal education are related to higher quality language input in the home (Hart and Risley, 1995). Specifically, higher maternal education is linked to children hearing more diverse words (Rowe, 2012), more complex syntax (Huttenlocher et al., 2002, Huttenlocher et al., 2007, Huttenlocher et al., 2010), and experiencing more conversational turns (Ferjan-Ramírez et al., 2020; Hirsh-Pasek et al., 2015; Romeo et al., 2018; Rowe, 2008; Schwab and Lew-Williams, 2016), all of which are linked to positive language outcomes. Similar to the relationship between stress and brain development, it is these daily language interactions that are thought to lead to differences in the development of language-specific brain regions in children whose mothers have lower levels of educational attainment.

In addition to variability in brain structure, maternal education and income differentially affect the development of cortical networks (Brito et al., 2020; Troller-Renfree et al., 2020; Pierce, Reilly and Nelson, 2021; Cantiani et al., 2019; Maguire and Schneider, 2019). Neurons in the human brain generate complex patterns of oscillatory activity defined as periodic and rhythmic shifting between high and low excitability states (Schroeder, Lakatos, Kajikawa, Partan, and Puce, 2008), all of which can be measured with resting-state EEG (rsEEG). Therefore, rsEEG is thought to measure the brain’s “readiness” to predict and integrate new experiences (Bice, Yamasaki and Prat, 2020). This ability to predict and integrate new experiences is essential to cognitive development, as it allows children to generate and test predictions about the world around them. Through consistent experiences with their environment these predictions are adjusted and shaped to allow them to generate the most accurate prediction. As these experiences accumulate over the course of development, they become ingrained in patterns of intrinsic resting-state brain function (Bice, Yamasaki and Prat, 2020). Taken together, aspects of a child’s environment that are intimately linked to SES, such as chronic physiological stress and the day-to-day language interactions children experience, are thought to have a cascading effect on functional brain development.

These differences in rsEEG support models of how maternal education and income influence brain development. Mothers who experience higher physiological stress have infants with increased relative power in low-frequency bands (theta) and reduced relative power in high-frequency bands (alpha, high-gamma; Troller-Renfree et al., 2020; 2023). These effects were observed in the whole brain (Troller-Renfree et al., 2020; 2023), but were most prominent in the frontal and parietal regions (Troller-Renfree et al., 2023). Importantly, poverty reduction interventions (e.g., paid maternal leave, universal income), result in decreases in relative low-frequency (Brito et al., 2022) and increases in relative high-frequency power (Brito et al., 2022, Troller-Renfree et al., 2022), as well as absolute power (Troller-Renfree et al., 2022), across all brain regions. Given chronic stress in the context of poverty can adversely affect the style of caregiving that parents provide (Blair and Raver, 2012, Blair and Raver, 2016, McLoyd, 1998), the reason poverty-reduction interventions may result in alterations in rsEEG may be attributed to an increase in engagement in enriching child activities during the first year of life (Magnuson et al., 2022) and a decrease in maternal depression through the pathway of decreased maternal stress (Ozer et al., 2011). Further, measures of high-quality language input have been associated with lower relative and absolute power in low-frequency bands (theta) and higher relative power in high-frequency bands (beta, gamma) in frontal regions and across infant brains using EEG (Brito et al., 2020, Pierce et al., 2021). Taken together, research has demonstrated that, higher family income and higher maternal education, may result in decreased rsEEG activity in lower frequency bands, but higher rsEEG activity in higher frequency bands because of differences in children’s everyday environments.

These individual differences in neural oscillations at rest matter, as rsEEG variations have been linked to specific mechanisms that subserve relevant cognitive outcomes, such as working memory (Andrews et al., 2021, Bhavnani et al., 2021, Swingler et al., 2011, Maguire and Schneider, 2019) and vocabulary knowledge (Lum et al., 2022; Meng et al., 2022; Maguire and Schneider, 2019; Cantiani et al., 2019; Benasich et al., 2008; Brito et al., 2016; Gou et al., 2011 . There is a general consensus among these studies that decreases in lower frequencies (theta) and increases in higher frequencies (alpha, beta, gamma) are positively associated with children’s working memory and expressive and receptive vocabulary skills. Importantly though, these patterns of decreased activation in lower frequencies and increased activation in higher frequencies are often not observed in infants and children from lower SES backgrounds (e.g., Shephard et al., 2019; Tomalski et al., 2013; Brito et al., 2020; Cantiani et al., 2019). Thus, higher family income and maternal education are linked to decreased activity in lower frequency bands and increased activity in higher frequency bands, both of which are reliable predictors of working memory and language outcomes.

Despite a clear association between rsEEG, maternal education, and income, no study to date has investigated the separable contributions of income and maternal education on rsEEG. Most studies examine SES as a single construct, collapsing across both variables, or examine only one of these indicators. Although income and maternal education are highly correlated, their role in child development is not the same. For example, consider a high school teacher who is a single-parent to three children—they would likely be considered lower income despite having a higher level of education, which could result in higher levels of physiological stress, while still providing their child with ample linguistic input and learning opportunities. By collapsing across income and maternal education, or only examining one construct, it is impossible to truly tease apart the unique impacts of each construct on child brain and language development. As a result, concrete, evidence-based practices to address the mechanisms that underlie these disparities, at an individual, school, or federal level, remain unattainable. Therefore, the current study examines the predictive nature of maternal education and income, independent of one another.

Furthermore, relatively few studies have examined these variables in school-aged children. This is problematic given it is the accumulation of experiences which exert their influence on patterns of intrinsic resting-state brain function. Infants, who are the focus of most rsEEG research, have significantly fewer accumulated experiences than older children. In addition, differences in neural oscillations at rest are linked to cognitive outcomes, making it critical that we understand how children’s neural activation relates to working memory and vocabulary knowledge as they are actively engaging these skills to progress through school.

In addition to the conventional univariate approaches, the current study also opted to use a machine-learning multivariate pattern analysis (MVPA) to identify which neural frequencies are most associated with SES and language processing skills. MVPA is sensitive to subtle differences in rsEEG data associated with a particular behavioral measure, by focusing on temporally and spatially distributed neural patterns rather than amplitude differences at a given time point in time in a given region of interest (Davis et al., 2014, Kriegeskorte et al., 2006, Jimura and Poldrack, 2012, Mur et al., 2009). In simpler terms, conventional univariate analyses commonly implemented in rsEEG studies measure the average activity in that region and correlates it with a specific behavior, whereas MVPA examines how the combined pattern of activity across different areas relates to a specific behavior. As such, MVPA may be able to delineate differential frequencies that vary on the basis of income and maternal education, independently, to predict working memory and vocabulary knowledge, which may be insensitive to conventional univariate methods.

## 2 Methods

### 2.1 Participants

One hundred ninety-five children were recruited from the Dallas-Fort Worth community. Data from 34 children (17.4 %) were discarded due to excessive artifacts in their rsEEG data (see EEG recording and preprocessing section below for more details). Therefore, a total of 161 children were included in the final dataset (*M* = 11.16 years; *SD* = 2.16; age range = 8–15 years; female = 90; Table 1). The age range of 8–15 years was examined, as this is a developmental period in which neural maturation is highly predictive of academic achievement in school (Sowell et al., 2004, Hashimoto et al., 2022). All children were right-handed as determined by the Edinburgh Handedness Inventory. Based on caregiver report, all children had no history of neurological disorders (e.g., cerebrovascular accident, seizure, traumatic brain injury) or learning disabilities. Maternal education level was based on self-report and income was based on eligibility for free and reduced lunch, which is determined based on national standards of income to needs ratio for families in the US (Mackey et al., 2015; Maguire and Schneider, 2019). The current sample was representative of the larger population from which it was drawn. According to a recent survey (U.S. Census Bureau, 2020), 10.9 % of families reported having less than a high school degree, which is comparable to 11.8 % in the current study. Similarly, 43 % of children in the United States qualify for free and reduced lunch, which was closely matched in the current study at a rate of 36.5 % (USA Facts, 2021). The distribution of age, maternal education level, and income across ages is reported in Table 2.

**Table 1:** Demographic profiles of participants.

	**N (%)**		**N (%)**
***Maternal Education***		***Income***	
Less than high school	19 (11.8 %)	High	102 (63.4 %)
High school degree	21 (13.04 %)	Low	59 (36.5 %)
Partial college	25 (15.5 %)	***Sex*** Male	73 (45.3 %)
College	60 (37.3 %)		
Graduate	36 (22.4 %)	Female	88 (54.7 %)
	***M*****(*****SD*****) [Range]**		***M*****(*****SD*****) [Range]**
***Child age at visit (year)***	11.2 (2.1) [8–15]	***Vocabulary (PPVT)***	111 (16.4) [71–152]
		***Working Memory (Digit Span)***	8.42 (2.61) [3–17]

**Table 2:** Distribution of Sex, Maternal Education, and Income across ages.

Variable		Age (in years)							
		8	9	10	11	12	13	14	15
Sex (*N*)	Male	9	8	9	14	10	10	8	5
	Female	11	11	15	16	9	9	13	4
Maternal Education (*N*)	Less than high school	4	1	2	2	4	2	3	1
	High school degree	4	0	2	4	3	1	4	3
	Partial college	3	3	4	6	2	3	3	1
	College	5	6	10	12	9	8	8	2
	Graduate	4	9	6	6	1	5	3	2
Income (*N*)	High	11	15	15	19	10	15	13	4
	Low	9	4	9	10	8	5	9	5

Children's language was assessed based on their receptive vocabulary score on the Picture Vocabulary Task 3rd or 4th Edition (PPVT; Dunn & Dunn, 1965). The PPVT-III and PPVT-IV were used interchangeably in this study since the PPVT was not used for clinical purposes, and because performance on both the PPVT 3rd and 4th editions is highly correlated (Spaulding & Hosmer, 2013). The reverse Digit Span, similar to that on the Wechsler Adult Intelligence Scale (WAIS; Wechsler, 2008), was used to assess working memory capacity. On this task, children heard number sequences increasing in length across trials and were asked to repeat the sequence back to the researcher in reverse order. Scores were based on the number of correct trials children completed, which was not standardized based on age.

### 2.2 Procedure

All participants were accompanied by a parent or primary caregiver and tested individually in a sound-attenuated and shielded room in a lab setting. Participants were seated in a chair one meter from a computer monitor and instructed to alternate between having their eyes open and closed as their EEG was recorded. The paradigm included four blocks of eyes-open and four blocks of eyes-closed conditions, alternating between each state for 1 minute each. A fixation cross appeared on the center of the screen to guide the participant's attention during the eyes-open condition, and the screen remained blank during the eyes-closed condition. A beep sound was played from a speaker as a cue to participants to open their eyes. Only data from the eyes-closed condition was used to reduce task demands and minimize visual input information (Anderson and Perone, 2018, Van Diessen et al., 2015). Written consent was obtained from parents, and verbal assent was obtained for the child participants following the University of Texas at Dallas IRB guidelines.

### 2.3 EEG recording and preprocessing

**Fig. 2:**
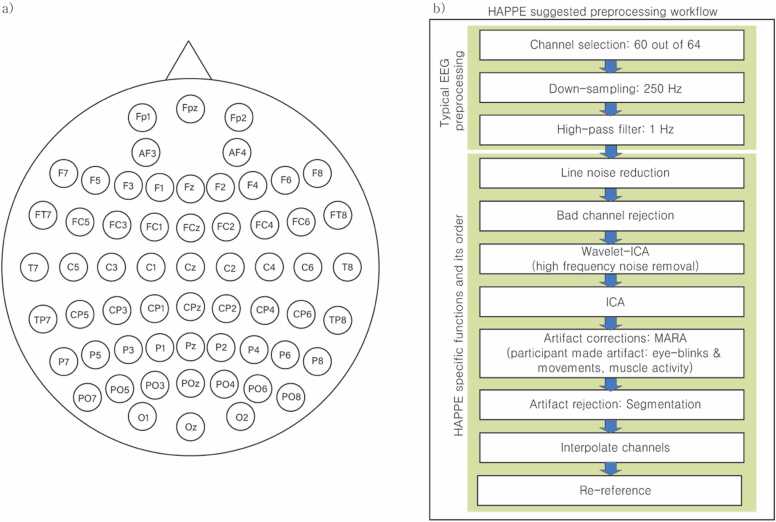
**A) Location of 60 EEG channels according to electrode placement scheme**: frontal pole (Fp), antero-frontal (AF), frontal (F), central (C), parietal (P), temporal (T), occipital (O), midline (z). Odd and even numbers indicate the left and right hemispheres, respectively. **B) The workflow of EEG preprocessing.** The HAPPE specific functions and order are combined with a standard preprocessing pipeline.

Impedances were kept below 10 kohms during EEG recording. Resting-state EEG was continuously recorded for 8 minutes using a 64-channel Quickcap and Neuroscan Synamp2 amplifier. The EEG data were collected at a sampling rate of 1000 Hz, high-pass filtered at 0.1 Hz, and low-pass filtered at 80 Hz via the CURRY Neuroimaging Suite Software. Sixty out of 64 channels from the eye-closed condition were used for data analysis (Fig. 2A). Preprocessing of raw EEG data was performed using EEGlab toolbox v14.1.1 (Delorme and Makeig, 2004) in Matlab 2017b (Mathworks, Inc.). In particular, we opted to use the Harvard Automated Processing Pipeline for Electroencephalography (HAPPE), a workflow optimized for children’s EEG data (Gabard-Durnam et al., 2018). In this preprocessing pipeline (Fig. 2B), raw data were first resampled at 250 Hz, and slow drifts were removed by a 1 Hz high-pass filter using a Hamming-window sinc FIR filter. Subsequently, several signal artifacts were removed including 60 Hz line noise reduction, bad channels rejection, pre-artifact correction via the wavelet-thresholding approach (Rong-Yi and Zhong, 2005). Next, independent component analysis (ICA) was performed and the Multiple Artifact Rejection Algorithm (MARA; Winkler et al., 2011) was used to remove noise artifacts associated with eye-blinks, movement, and muscle activity. Additionally, epoch-based artifact rejection was applied by segmenting the 4-minute-long eyes-closed EEG data into 2-second bins. Spherical interpolation was applied to those rejected bad channels prior to re-referencing of all EEG data to the average of all channels. After preprocessing, 34 children’s EEG data were excluded due to an excessive amount of rejected segmentation (> 30 %; *N*=3), too many rejected channels (>30 %; *N*=18), too many rejected independent components (>50 %; *N*=10), and an insufficient amount of raw EEG (*N*=3).

### 2.4 EEG analysis using machine-learning pattern regression

After preprocessing, we performed a series of machine-learning analyses to predict PPVT and maternal education (Table 1) among all 161 children, and Digit Span among a subset of 131 children. This analysis was conducted using scikit-learn, a python-based platform (http://scikit-learn.org/stable). We opted to use support vector regression (SVR; Drucker et al., 1996) with a Radial Basic Functional (RBF) kernel of a linear support vector machine (SVM; Ding et al., 2021). To this end, the continuous EEG signals were Fourier-transformed to the absolute power spectrum densities (PSDs) and averaged at every channel per each of the following frequencies: Theta (4–6 Hz), Alpha (7–12 Hz), Beta (13–19 Hz), Lower Gamma (21–30 Hz), and Higher Gamma (31–45 Hz). For low-to-mid frequency ranges, we referred to Vanderwert et al. (2016), who also examined school-age children; however, because this study did not include high frequency ranges, we referred to Tomalski et al. (2013)’s infant EEG study to specify high frequency ranges (Table 3). In addition to the absolute power, we also calculated the relative power. This was done by first taking the average across all frequency bands spanning from 4 to 45 Hz. We then took the average of each frequency band (e.g., average across 4–6 Hz for Theta) before dividing the average of a particular frequency (e.g., theta) by that of the entire frequency range.

**Table 3:** Frequency bands examined in the current study based on previous research in children.

Frequency ranges (Hz)						
Current study			Vanderwent et al. (2010)		Tomalski et al. (2013)	
Theta	4–6	Theta		4–6	Theta	3–5
Alpha	7–12	Alpha		7–12	Alpha	6–9
Beta	13–20	Beta		13–20	Beta	13–19
Lower Gamma	21–30				Lower Gamma	21–30
Higher Gamma	31–45				Higher Gamma	31–45

These PSD data were then submitted to SVR. Given age was significantly predicted by absolute PSD in all frequency bands (Supplementary Table 1), all models included age (normalized z-scores) as a covariate. Sex was also included as a binary variable in all models (Etchell et al., 2018, Kolb et al., 1998). The SVR models underwent 10-fold cross-validation, in which 90 % of data were randomly selected for a training set, and the remaining 10 % were used for a testing set per each fold. To find the ideal RBF hyperparameters, we used grid-search-cross validation hyperparameter tuning which implemented a brute force method (Kumar et al., 2021). Consequently, the level of curvature (gamma), the error penalty parameter (C values), and the width of the hyperplane (epsilon) were determined to yield the best R^2^, a score reflecting the degree of SVR’s model fitness. The significance of the model fitness was evaluated via a Monte Carlo simulation with 10,000 iterations, in which a null distribution was generated by randomly shuffling the labels of input vectors (Supplementary Figure 1). The *p*-value was further adjusted for multiple comparisons using the Bonferroni’s correction at *p* <.05. Further, due to the highly correlated relationship between PPVT, Digit Span, maternal education, and income (Table 4), we performed SVR analysis on the PPVT, maternal education, and income after residualizing confounding variables from the rsEEG data (Snoek et al., 2019). For instance, rsEEG data were regressed against PPVT and income before SVR on maternal education. Similarly, for PPVT, a regression was performed after regressing out maternal education and income from rsEEG. Due to a different number of participants (*N* = 131), the Digit Span was not regressed out. Given maternal education and income were not significantly correlated with the Digit Span, but PPVT scores were (see Table 4), only the PPVT was regressed out for SVR on the Digit Span.

**Table 4:** Correlation results between demographic variables and vocabulary score.

Variables	Age	Sex	Maternal Education	Vocabulary (PPVT)	Income	Working Memory (Digit Span)
Age						
Sex	0.002					
Maternal Education	−0.091	0.105				
Vocabulary (PPVT)	−0.082	0.148	0.520***			
Income	0.052	−0.160	0.477***	0.687***		
Working Memory (Digit Span)	0.032	0.120	0.151	0.245***	0.141	

*Note. P* <0.001***.

### 2.5 EEG analysis using machine-learning pattern classification

In addition, we performed machine-learning classification using a support vector machine (SVM) to decode high vs. low-income status from rsEEG data. We used a stratified Leave-P-Out cross-validation procedure, in which P refers to “proportion” and the number of folds was determined based upon the smallest class size (i.e., 59 folds cross-validation). In the current study, the Leave-P-Out cross-validation uses stratified sampling to ensure that the proportion of high- vs. low-income status is preserved across training set and test set. For instance, if the class distribution stands at 1:2 for Class A (low income) and Class B (high income), respectively, one instance from Class A and two instances from Class B would be designated for the testing set, while the remaining data points would constitute the training set. This methodology ensures that the ratio of classes is preserved within both training and testing subsets, promoting equitable representation for accurate model assessment.

To find the ideal RBF hyperparameters of the model’s performance, we applied a grid-search-cross-validation by tuning the level of curvature (gamma) and the error penalty parameter (C values). Because of the unbalanced participant numbers between high- and low-income status (see Table 1), the model’s prediction could be biased by the class with the largest number. This problem was handled by using an F1-score, the weighted average of the precision (the number of true positives divided by the sum of true positives and false positives) and recall (the number of true positives divided by the number of false negatives; Van Fijsbergen, 2004). We also penalized the algorithm that increased the cost of misclassification of the minority class. A Monte Carlo simulation (with 10,000 iterations) was performed to determine the significance of the F1-score, which was adjusted for multiple comparisons across all frequency bands using Bonferroni correction. Again, due to the highly correlated relationship between income and our other variables (PPVT, maternal education, Digit Span; Table 4), we performed SVM classification analysis on income after residualizing PPVT, maternal education (Snoek et al., 2019). However, Digit Span was not residualized due to differences in sample size. Age and sex were also included as covariates, similar to the SVR analysis.

### 2.6 EEG Analysis using conventional multiple regression and correlation analyses

In addition, conventional univariate multiple regression and correlation analyses were performed between PSD (independent variable) and PPVT, Digit Span, maternal education, and income (dependent variables) at every channel. Ordinary least square (OLS) in statsmodel, a python-based platform (http://statsmodel.org/stable) was used for multiple regression analysis, in which PSD of 60 channels, age, and sex were included as predictors. We employed a residualization approach for univariate multiple regression analyses too. For example, when predicting the PPVT variable, the independent variables consisted of absolute PSD from 60 channels. The dependent variable was residualized PPVT scores, obtained by regressing out the influences of income, sex and age, similar to methods used in other MPVA analyses. Model regression coefficients and their *P*-values were calculated for each of the 5 frequency bands. Due to high multicollinearities in predictor variables, PSDs and age variables were normalized to z-scores. We used a Pearson’s correlation in scipy.stats (http://docs.scipy.org/doc/scipy), a python-based platform to compute correlation analysis. As PSD violated the normality assumption, we used a non-parametric permutation approach for testing *P*-values. Correlation coefficients and corresponding *P*-values were computed for each of the 5 frequency bands. All *P*-values from the univariate analyses were further corrected for the multiple comparisons via the Bonferroni’s correction at *p* <.05.

## 3 Results

The current study sought to identify which frequencies of neural oscillations at rest, as measured with EEG, are sensitive to unique aspects of a child’s environment (e.g., income and maternal education) and how fluctuations in these oscillations relate to cognitive and language processing skills (as measured by vocabulary and working memory). Since vocabulary knowledge (PPVT), working memory capacity (Digit Span), and maternal education were continuous variables, we conducted support vector regression (SVR) analyses. Analyses including vocabulary knowledge and maternal education were run on the full sample of 161 children, while analyses including working memory were run on a subset of 131 children. Although a few studies investigating the impacts of early life experiences on brain function have reported absolute PSD (e.g., Troller-Renfree et al., 2022; Brito et al., 2020), relative PSD is most commonly reported. For this reason, we elected to run our analyses using both absolute and relative PSD. Similar to Brito et al., (2020), our analysis of relative PSD yielded primarily null results (see Supplementary Tables 2 and 3). The only relationship to withstand Bonferroni corrections was between high gamma and maternal education.

We also examined the relationship between absolute PSD and the PPVT, Digit Span, and maternal education. Table 5 summarizes these SVR models’ R^2^ scores and their *P*-values. Note that the negative R^2^ indicates a failure of SVR model-fitting (Iizuka et al., 2020, Xiong et al., 2021) and only the positive R^2^ were further evaluated for significance testing. As a result, we found that beta and lower gamma were predictive of PPVT scores, theta was predictive of Digit Span scores, and alpha and lower gamma were associated with maternal education.

**Table 5:** SVR model results of predicting PPVT, Digit Span and Maternal Education, absolute PSD.

Target	Residualized variables	Predictors	Frequency bands	R^2^ score	*P*-value	Adj. *P*-value
PPVT	Maternal Education + Income	rsEEG + Age + Sex	Theta	−0.029	-	-
			Alpha	−0.026	-	-
			Beta	0.007	0.005	0.012*
			Lower Gamma	0.030	0.001	0.002**
			Higher Gamma	−0.025	-	-
Maternal Education	PPVT + Income	rsEEG + Age + Sex	Theta	−0.006	-	-
			Alpha	0.011	0.004	0.008**
			Beta	−0.004	-	-
			Lower Gamma	0.048	0.001	0.001***
			Higher Gamma	−0.007	-	-
Working Memory	PPVT	rsEEG + Age + Sex	Theta	0.049	0.002	0.002**
			Alpha	−0.008	-	
			Beta	−0.008	-	
			Lower Gamma	−0.015	-	
			Higher Gamma	−0.015	-	

*Note.* *** *P* <.001, ** *P* <.01, * *P* <.05. rsEEG: resting state EEG. Adj.: Adjusted using Bonferroni correction. -: Not applicable. Age and sex were included as covariates across all models. Due to the collinearity of different variables as reported in Table 4, we also residualized PPVT when predicting maternal education and working memory, income when predicting maternal education and PPVT, and maternal education when predicting PPVT.

Income was included as a binary measure (i.e., high vs low), which was suitable to the MVPA classification using support vector machine (SVM) learning to decode high vs. low-income status from absolute power rsEEG data. This analysis was run on the full sample of 161 children. Table 6 summarizes the SVM models’ F1 scores and *p*-values for income. Theta and alpha power reliably predicted the income status of children’s families.

**Table 6:** SVM model results of classifying Income, absolute PSD.

Target	Residualized variables	Classifiers	Frequency bands	F1 score	*P*-value	Adj. *P*-value
Income	PPVT + Maternal Education	rsEEG + Age + Sex	Theta	63.2	0.001	0.005**
			Alpha	60.5	0.005	0.026*
			Beta	54.6	0.069	0.345
			Lower Gamma	57.3	0.024	0.119
			Higher Gamma	55.3	0.062	0.311

*Note.* ** *P* <.01, * *P* <.05. rsEEG: resting state EEG. Adj.: Adjusted using Bonferroni correction. -: Not applicable. Age and sex were included as covariates. Due to the collinearity of different variables as reported in Table 4, we also residualized PPVT and maternal education when predicting Income.

Conventional univariate approaches including regression and correlation were conducted between PSD (independent variable) and PPVT, Digit Span, maternal education, and income (dependent variables) at every channel. Both conventional approaches failed to yield any significant results (Supplementary Table 4 & Supplementary Figure 2).

Furthermore, SVR and SVM analyses without residualization were conducted for the PPVT, maternal education, and income to discern potential overlapping information between these variables. The results revealed that both theta and alpha frequencies significantly predicted PPVT and maternal education, while both lower and higher gamma frequencies significantly predicted income and maternal education (see Supplementary Tables 5 & 6). These findings indicate that our residualization approach adopted a more conservative stance in associating specific rsEEG signal frequencies with distinct aspects of PPVT, maternal education, and income.

## 4 Discussion

Previous research has reported a relationship between unique aspects of SES and infant brain and cognitive development; however, SES in and of itself is not the cause of neural and cognitive differences. Instead, SES is a multifaceted measure that acts as a proxy for differences in children’s home environments, the effects of which accumulate throughout development, impacting subsequent brain, language, and cognitive development. In the current study we sought to better understand the nature of these behavioral and neural differences by examining the rsEEG of school-aged children (ages 8–15 years) in relation to the unique contributions of maternal education and income. Using MVPA we were able to identify that both constructs of SES are linked to neural function in the alpha frequency (7–12 Hz), but that two dissociable relationships for each construct emerge. The first relationship is between maternal education, lower gamma (21–30 Hz) activity, and vocabulary outcome. The second relationship is between income, theta (4–6 Hz) activity, and working memory ability. Importantly, while there is some neuroimaging evidence that income and parental education have differential effects on neural structure (Noble et al., 2015, Simon et al., 2021), we expand upon this research by showing that income and maternal education also have differential effects on resting-state neural oscillations in older children and that those neural differences are related to language and cognition. Identifying the unique pathways by which constructs of SES exert their influence on subsequent brain, language, and cognitive development can ultimately help to explain how environmental experiences may influence the developing brain, and subsequent language and cognitive outcomes.

A unique and important contribution of the current study is that maternal education and income have dissociable influences on subsequent brain and behavioral outcomes. Specifically, maternal education corresponds to differences in high frequency lower gamma activity and vocabulary outcome, while income variability is related to differences in low frequency theta and working memory ability. This approach diverges from previous studies which either used a composite score combining maternal education and household income into a single metric or used only one SES factor (i.e., income or maternal education). Our approach is not meant to replace those approaches which have been instrumental in advancing the field of developmental cognitive neuroscience, but to add to our knowledge by demonstrating there are also dissociable relationships between income and maternal education on child brain development. For example, not including a composite measure of SES in the current study could be seen as limiting our ability to assess the broader influence of SES on child outcomes in a single, simplified score, however, it offers a distinct advantage. By examining maternal education and income separately, this study is able to capture the unique contributions of each factor, acknowledging that different aspects of SES can affect development in varying ways (Bornstein and Bradley, 2014, Shavers, 2007). This approach provides a nuanced understanding of the relationship between aspects of SES and child outcomes, avoiding the potential masking of individual effects that composite measures can sometimes cause (Darin-Mattsson et al., 2017, Long and Renbarger, 2023). Notably, we found that differences in alpha were in fact related to both maternal education and income, potentially capturing some of the broader influences of SES on brain development. Similarly, even with this unique approach, our findings support those of structural MRI studies that focus on individual SES measures, which demonstrated that parental education level, but not income, accounted for SES related variance in amygdala volume, the part of the brain associated with socioemotional functioning (Noble et al., 2012). Meanwhile, income, but not parental education level, accounted for significant variance in hippocampal volume, the part of the brain that is related to memory functioning (Noble et al., 2015). Given SES is most often measured by educational attainment and income, but rarely in the same analysis, or as a unitary measure, it is critical that future research considers the dissociable effects of both income and maternal education on children’s functional and structural brain development.

The current study brings together past research findings by identifying a link between lower gamma, maternal education, and vocabulary ability. Differences in rsEEG lower gamma related to SES have been uncovered as early as infancy (Pierce et al., 2021; Cantiani et al., 2019; Troller-Renfree et al., 2022). Pierce et al. (2021) reported that a greater number of vocalizations and conversational turns, both of which are positively correlated with maternal education, were also associated with higher relative gamma power. Fluctuations in gamma activation are also thought to underlie the concurrent development of neural networks that support emerging language and cognitive skills (Benasich et al., 2008; Crone et al., 2001; and see Tomalski et al., 2013 for discussion). Specifically, rsEEG lower gamma has been related to language abilities in young (15–36 months of age; Benasich et al., 2008; Brito et al., 2016; Cantiani et al., 2019) and older children (ages 7–9 years). In older children, lower gamma, referred to as beta in that study, was predictive of later receptive vocabulary knowledge (Meng et al., 2021). Although the current study does not include longitudinal measures, thereby limiting our ability to make causal claims, it appears that the neural networks supported by lower gamma may be shaped by aspects of children’s environments that are linked to maternal education, such as the home language environment. These same lower gamma fluctuations are then highly predictive of children’s vocabulary skills during the school years.

Fluctuations in resting state lower gamma were not the only frequency associated with language outcome, fluctuations in beta power also held a significant relationship with vocabulary knowledge. The beta frequency band is one of the primary frequencies engaged in cortico-basal ganglia loops (e.g., Brittain, Sharott, and Brown, 2014; Stein and Bar-Gad, 2013), which are thought to play an important role in language processing (e.g., Booth et al., 2007; Kotz, Schwartze, and Schmidt-Kassow, 2009; Lewis et al., 2023; Kim et al., 2024). In task-based EEG studies, beta is related to a number of language processes, including action semantics, semantic and syntactic congruency, semantic binding, and semantic memory (for review, see Weiss and Mueller, 2012). Our findings establish that the relationship between resting state beta power and language processing exists as early as 8 years old. It is also not surprising that resting state beta was not associated with aspects of SES, as only one study to date has reported an association between environmental experiences and beta. In that study, 6- to 12-year-old children from lower SES backgrounds, defined as very low income and/or having an illiterate mother, had higher values of absolute power across all frequency bands, including beta (Harmony et al., 1990). This study however did not consider the role of language, leaving open the possibility that SES differences in beta power may have been the result of SES based differences in vocabulary knowledge. The relationship between high frequency oscillations like beta and gamma and language in the current study make sense based on past research. High frequency beta and gamma activity facilitate the functional networks required for the binding or integration of information, such as the temporally distributed auditory signals that make up language (e.g., Csibra et al., 2000; Fries, 2009). Therefore, while maternal education is related to resting state lower gamma, high frequency oscillations more broadly have an integral role in vocabulary development.

The association between rsEEG theta, income, and working memory uncovered in the current study is also heavily tied to past research. Early adverse life events, linked to income, physiological stress, and neglect, have been associated with alterations in resting state theta. Specifically, among individuals who experienced significant psychosocial adversity early in life compared to those who did not, higher posterior theta has been observed from infancy (Marshall et al., 2004; Pierce et al., 2021; Cantiani et al., 2019; Troller-Renfree et al., 2022) through age 16 (Debnath et al., 2020; Maguire and Schneider, 2018). The current study adds to these past research findings, identifying income, but not maternal education, as a predictor of higher theta power at rest. Numerous studies have also advocated for the role of theta power in working memory tasks (Klimesch, 1999, Gevins et al., 1998, Raghavachari et al., 2001, Raghavachari et al., 2006, Böcker et al., 2010). Related to rsEEG theta, in a sample of school-aged children, Maguire and Schneider (2019) found that increases in theta power at rest significantly predicted working memory. Importantly, children from higher income homes were more likely to demonstrate increases in theta at rest. In conjunction with the current findings, it appears that early life experiences tied to income and working memory performance may differentially shape the neural networks underlying theta activation.

The current MVPA analyses employed failed to replicate all of the absolute PSD results when examining differences in relative PSD. We did uncover a significant relationship between higher relative gamma PSD and maternal education, similar to our significant relationship between lower absolute gamma PSD and maternal education. Previous studies examining differences in lower and higher gamma often collapse across both frequency bands, making it difficult to interpret whether lower and higher gamma changes in absolute and relative PSD relative to maternal education are dissociable or the same. For example, Cantiani et al. (2019) reported that oscillatory gamma activity, from 25 to 45 Hz, mediates the pathway from maternal education to language acquisition in infancy. Therefore, it appears that the relationship between maternal education and gamma power holds across the entire frequency range and across absolute and relative PSD. It may also not be so surprising that many of our findings based on absolute PSD were not significant when examining changes in relative PSD. In a study implementing conventional univariate analyses to examine the relation between the home language environment and rsEEG in infants, Brito et al. (2020) found that absolute PSD, but not relative PSD, yielded significant results. Furthermore, most, if not all, rsEEG studies utilizing MVPA exclusively examine changes in AP (Jach et al., 2020, Golemme et al., 2021, Bae and Luck, 2018). Another reason for the lack of replication across absolute and relative PSD pertains to the wide age range (8–15 years) examined in the current study. Across this developmental window, the brain is undergoing substantial changes associated with maturation, indicating the absolute power of rsEEG signals can be drastically different between pre-adolescence and adolescence. Given this weaker and more conservative relative PSD result, we speculate that the dynamic nature of rsEEG signals was obscured by converting the data to the relative scale, which yielded much narrower distance among the data points in the multi-dimensional space. In other words, absolute power may better characterize distinct EEG signals pertaining to different ages of children in this study, as has been shown in past research. Future research is certainly warranted when exploring rsEEG among adolescents to corroborate our initial findings.

Taken together, the current findings provide evidence that components of SES have differential associations with brain function and related cognitive processes. Our data point to dissociable pathways in which maternal education is linked to fluctuations in resting state lower gamma and vocabulary outcome, while income is associated with resting state theta and working memory ability. Interestingly though, changes within the alpha band were related to both aspects of SES. Alpha activity is highly susceptible to individual differences in a number of cognitive processes, including processing speed (Klimesch et al., 1996) , intelligence (e.g., Anokhin and Vogel, 1996; Doppelmayr, Klimesch, Stadler, Pöllhuber, and Heine, 2002), attention (Klimesch, Schimke, and Pfurtscheller, 1993), language processing (Bice et al., 2020, Bornkessel et al., 2004), and inhibitory control (Klimesch et al., 2007; Strauß, Wöstmann, Obleser, 2014). In general, higher alpha power at rest and lower alpha power during tasks are related to better cognitive function and task performance; however, greater increases in alpha power also result in better performance when the task at hand is more difficult. This paradox has been reconciled with an inhibitory account of alpha function (Cooper et al., 2003, Jensen and Mazaheri, 2010; Klimesch et al., 2007), such that alpha activity is related to attentional control and inhibition of irrelevant cortical activity. Based on this inhibitory account, we speculate that more global aspects of SES, related to both maternal education and income, impact alpha activation at rest, which may in turn have downstream effects on inhibitory control, which was not measured in the current study.

A strength of the current study was the use of machine learning multivariate pattern-based analyses (MVPA). This method has been extensively applied to fMRI data (Tong and Pratte, 2012, for a review), wherein differential patterns of BOLD (blood oxygen-level dependent) activity across multiple voxels are used as features to contain information related to different conditions (Pereira et al., 2009) that may be comparable in overall amplitude. Similarly, EEG utilizes time course signals from multiple electrodes (Grootswagers et al., 2017) and differential patterns of neural activity across electrodes at a given frequency range (e.g., beta, lower gamma, etc.) to convey unique information pertaining to behavioral measures. In fact, many neuroimaging studies now report results using both MVPA and conventional univariate analyses, with the goal of drawing different conclusions from each. This is because MVPA is sensitive to latent, multidimensional neural representations, while conventional univariate approaches are not. Thus, differences in activation patterns across both analysis techniques are due to the fact that MVPA can better handle the multidimensional nature of neural data. This was precisely the case with our current results in that we were able to identify a set of frequencies associated with different aspects of SES and behavioral outcomes when performing MVPA, while conventional univariate analysis failed to detect these same effects. To date, EEG researchers have used MVPA to decode a range of neural correlates of perceptual and cognitive processes, including perceptual decision making (Bode et al., 2012), sensory representation (Hogendoorn et al., 2015), face detection (Cauchoix et al., 2014), sustained attention and working memory (Bae, Luck, 2018), and grammar ability (Zhao et al., 2021) . To the best of our knowledge, this is the first application of MVPA to school-age children’s rsEEG data, demonstrating how dissociable frequency ranges relate to income and maternal education.

It is important to acknowledge that, while a wealth of research has linked maternal education to variability in the home environment and income to physiological stress, we do not explicitly measure these environmental experiences in the current study. Our conclusions about the role of the home language environment and physiological stress, despite being rooted in past research, are speculatory. Future directions involve directly measuring environmental experiences to strengthen the current model of how dissociable aspects of SES influence child brain and language development. Due to a difference in the amount of digit span data available (a proxy of working memory), we could not include this as a covariate in models predicting vocabulary (as measured by the PPVT) using SVR. We acknowledge that there may be a potential influence of the digit span on the model with vocabulary, given they were positively correlated. The reason for this difference in data points relates to the fact that this dataset is part of a larger study (Maguire et al., 2018), which integrated the Digit Span into the battery of behavioral assessments partway through data collection. Similarly, only 36.5 % of the current sample came from a low-income household, with 11.8 % of families reporting their education as less than a high school diploma. Although this is representative of the larger United States, a more even distribution of low- to high-income families may yield different results. Another important limitation of the current study is that we investigate the relationship between aspects of a child’s SES, brain, language, and cognitive development at a single point in time. Another important limitation of the current study is that we investigate the relationship between aspects of a child’s SES, brain, language, and cognitive development at a single point in time across a wide age range. Although age was controlled for in all models, robust increases in rsEEG occur across the course of development, with the most prominent changes occurring from pre-teen to adolescence, primarily in fronto-central regions (van Noordt and Willoughby, 2021). Therefore, the associations between rsEEG, SES, and language/cognitive development in the current study may vary across the course of the school years. Given the accumulation of environmental experiences over time is what impacts brain, language, and cognitive development, future longitudinal research should examine how the pathways between these factors vary over the course of development. It would be particularly interesting to bridge the gap between existing rsEEG studies in infancy with the current study of school-aged children to better establish how the accumulation of very early life experiences impacts the vocabulary and working memory skills of children as they progress through school.

The results of the current study do not indicate that children from lower SES homes experience a maturational lag or deficit in neural development, but rather that discrete neural frequencies are engaged differentially on the basis of income and maternal education. In fact, children who experience enhanced adversity may exhibit strengths in skills and abilities that help them meet real-world challenges in their environments, referred to as hidden talents (i.e., The Hidden Talents Framework; Ellis et al., 2017, Ellis et al., 2020; Frankenhuis and de Weerth, 2013; Frankenhuis et al., 2020). For example, individuals who experience unpredictable environments have been shown to be more efficient at shifting attention and more accurate when responding on working memory updating tasks (Fields et al., 2021, Nweze et al., 2021, Young et al., 2022). These same results are apparent in the current study, as working memory performance was not related to either income or maternal education, and thus lower SES children may be demonstrating strengths in working memory ability. Although both measures of SES were correlated with performance on the PPVT, there is some evidence that this vocabulary assessment may be biased against culturally and linguistically diverse populations (Haitana et al., 2010; Stockman, 2000) , especially those from low-income backgrounds. Although similar SES-based vocabulary differences have been observed utilizing other standardized measures (Levine et al., 2020, Fernald et al., 2013), natural language samples (Schneider et al., 2022, Huttenlocher et al., 2007, Walker et al., 1994) and measures of word learning (Kelley and Bueno, 2022; Shavlik et al., 2021; Maguire et al., 2018), the utilization of the PPVT in the current study could have overinflated differences in vocabulary knowledge on the basis of SES. In summation, the neural differences observed here likely demonstrate differences in neural adaptability, rather than a delay or deficit, associated with environmental experiences.

In 2023, over 11 million children were living below the federal poverty line in the United States, placing it as the third highest child poverty rate among OECD countries (Organization for Economic Co-operation and Development). These children are at a heightened risk for falling behind academically due to environmental factors that have detrimental effects on their subsequent mental, cognitive, and physical health. Language processing is among the most negatively affected domains (Perkins, Finegood and Swain, 2013), which is problematic given language skills are the strongest predictor of subsequent academic success (Pace et al., 2019). Therefore, it is of critical societal and economic importance that we better identify how different aspects of a child’s environment, tied to income and maternal education, independently influence brain, language, and cognitive development in school-aged children. Here we show dissociable pathways by which income and maternal education may exert their influence on children’s subsequent language processing outcomes.

## Funding

This work was supported by the National Science Foundation Developmental Sciences Division under Grant 1551770 (PIs: Maguire and Abel).

## CRediT authorship contribution statement

**Yune S. Lee:** Writing – review & editing, Supervision, Formal analysis. **Sonali Poudel:** Writing – review & editing. **Jeahong Kim:** Writing – review & editing, Formal analysis. **Julie M. Schneider:** Writing – review & editing, Writing – original draft, Supervision, Methodology, Conceptualization, Data curation. **Mandy J. Maguire:** Writing – review & editing, Writing – original draft, Supervision, Project administration, Methodology, Funding acquisition, Data curation, Conceptualization.

## Declaration of Competing Interest

The authors declare that they have no known competing financial interests or personal relationships that could have appeared to influence the work reported in this paper.

## Data Availability

Data will be made available on request.
