# Supplementary material for: Socioeconomic status (SES) and cognitive outcomes are predicted by resting-state EEG in school-aged children
Source: Dev Cogn Neurosci. 2024 Oct 29;70:101468. doi: 10.1016/j.dcn.2024.101468 (PMC11570756; doi:10.1016/j.dcn.2024.101468)
Supplement: Supplementary Table 1 — Supplementary material. [file mmc1.docx]

**5. Supplementary Material**

| 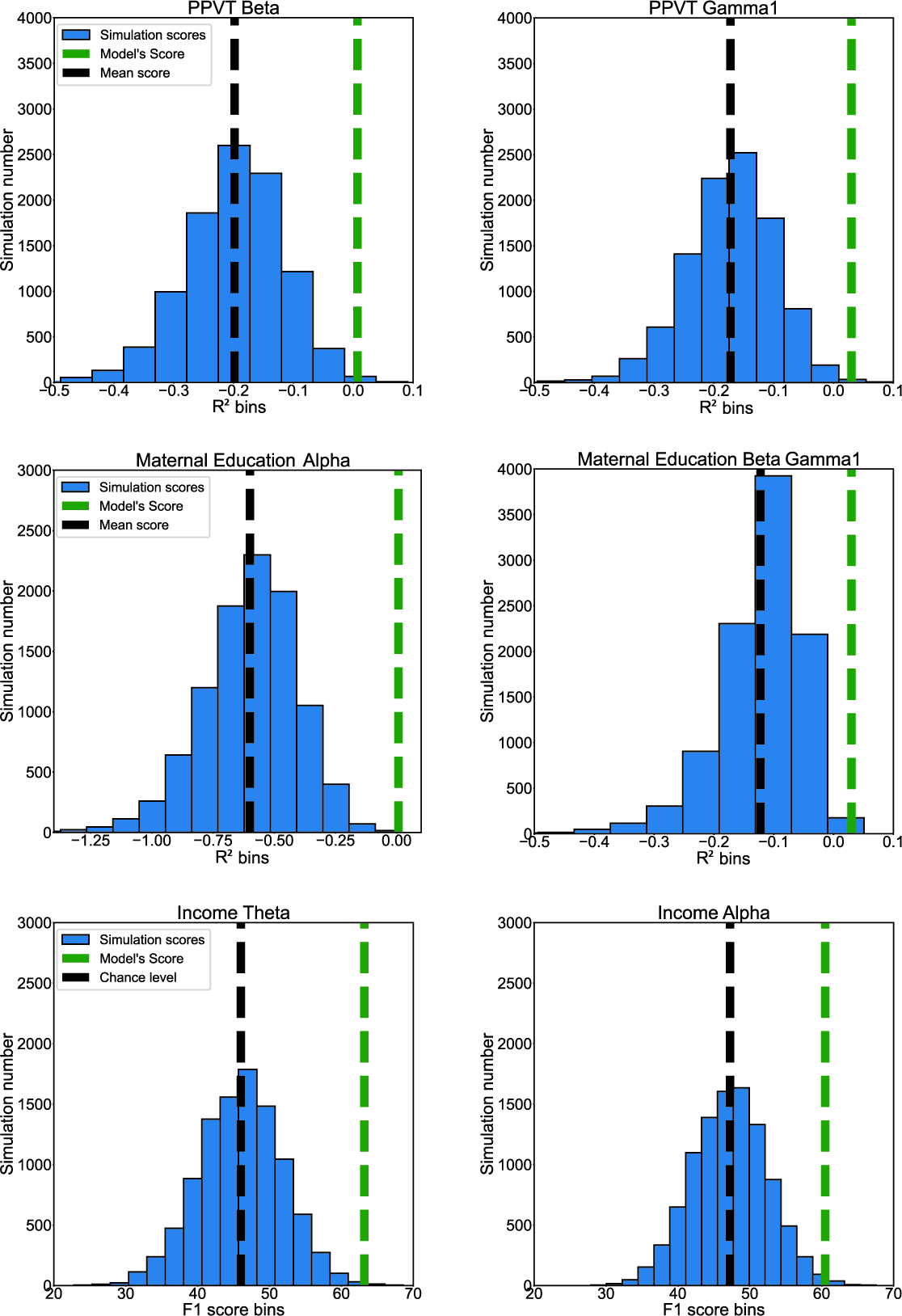  **Supplementary Figure 1. Significance testing of support vector models using Monte Carlo simulation.** The top row shows simulation distribution for PPVT, the middle row shows maternal education, and the bottom row shows income. Green dotted line: model’s performance score from correct labeling. Black dotted line: Mean score of the randomly distributed performance. Blue bars: histogram frequencies of performance score obtained from 10,000 analysis using randomly shuffled data. |
| --- |

**Supplementary Table 1. SVR model results of predicting Age.**

| Target | Predictors | Frequency bands | R^2^ score | *P*-value | Adj. *P*-value |
| --- | --- | --- | --- | --- | --- |
| Age | rsEEG + Sex | Theta | 0.083 | 0.001 | 0.005 ** |
|  |  | Alpha | 0.007 | 0.002 | 0.010 * |
|  |  | Beta | 0.047 | 0.001 | 0.005 ** |
|  |  | Low Gamma | 0.085 | 0.001 | 0.005 ** |
|  |  | High Gamma | 0.079 | 0.001 | 0.005 ** |
| *Note.* ** *P* < .01, * *P* <.05. rsEEG: resting state EEG. Adj.: Adjusted using Bonferroni correction. Sex was included as a covariate. | | | | | |

Supplementary Table 2. SVR model results of predicting PPVT, Digit Span and Maternal Education, relative PSD.

| Target | Residualized variables | Predictors | Frequency bands | R^2^ score | *P*-value | Adj. *P*-value |
| --- | --- | --- | --- | --- | --- | --- |
| PPVT | Maternal Education +  Income | rsEEG +  Age +  Sex | Theta | -0.079 | - | - |
|  |  |  | Alpha | -0.078 | - | - |
|  |  |  | Beta | -0.076 | - | - |
|  |  |  | Low Gamma | -0.079 | - | - |
|  |  |  | High Gamma | -0.068 | - | - |
| Maternal Education | PPVT +  Income | rsEEG +  Age +  Sex | Theta | -0.050 | - | - |
|  |  |  | Alpha | -0.050 | - | - |
|  |  |  | Beta | -0.047 | - | - |
|  |  |  | Low Gamma | -0.050 | - | - |
|  |  |  | High Gamma | 0.026 | 0.001** | 0.001** |
| Working Memory | PPVT | rsEEG +  Age +  Sex | Theta | -0.057 | - | - |
|  |  |  | Alpha | -0.066 | - | - |
|  |  |  | Beta | -0.082 | - | - |
|  |  |  | Low Gamma | -0.095 | - | - |
|  |  |  | High Gamma | -0.097 | - | - |
| *Note.* *** *P* < .001, ** *P* < .01, * *P* <.05. rsEEG: resting state EEG. Adj.: Adjusted using Bonferroni correction. -: Not applicable. The same residualization and covariates were included in models examining absolute PSD were utilized in the current analyses. | | | | | | |

Supplementary Table 3. SVM model results of classifying Income, relative PSD

| Target | Residualized variables | Classifiers | Frequency bands | F1 score | *P*-value | Adj. *P*-value |
| --- | --- | --- | --- | --- | --- | --- |
| Income | Maternal Education | rsEEG +  Age +  Sex | Theta | 49.3 | 0.421 | 1 |
|  |  |  | Alpha | 55.2 | 0.068 | 0.340 |
|  |  |  | Beta | 48.9 | 1 | 1 |
|  |  |  | Low Gamma | 50.5 | 0.247 | 1 |
|  |  |  | High Gamma | 57 | 0.029* | 0.145 |
| *Note.* ** *P* < .01, * *P* <.05. rsEEG: resting state EEG. Adj.: Adjusted using Bonferroni correction. -: Not applicable. Maternal education was residualized, and age and sex were included as covariates, similar to the previous analysis examining relative PSD. | | | | | | |

Supplementary Table 4. Summary of multiple regression analyses results after residualizing confounding variables

| Dependent Variable | Frequency | R^2^ | Adj. R^2^ | *F* change (Adj. *P*) |
| --- | --- | --- | --- | --- |
| PPVT | Theta | 0.222 | -0.215 | 0.392 (*0.999*) |
|  | Alpha | 0.219 | -0.218 | 0.454 (*0.999*) |
|  | Beta | 0.200 | -0.337 | 0.474 (*0.999*) |
|  | Lower Gamma | 0.213 | -0.324 | 0.421 (*0.999*) |
|  | Higher Gamma | 0.202 | -0.335 | 0.405 (*0.999*) |
| Maternal Education | Theta | 0.258 | -0.220 | 0.457 (*0.943*) |
|  | Alpha | 0.239 | -0.252 | 0.563 (*0.998*) |
|  | Beta | 0.224 | -0.276 | 0.550 (*0.992*) |
|  | Lower Gamma | 0.267 | -0.201 | 0.492 (*0.997*) |
|  | Higher Gamma | 0.252 | -0.226 | 0.548 (*0.984*) |
| Income | Theta | 0.266 | -0.232 | 0.555 (*0.999*) |
|  | Alpha | 0.243 | -0.254 | 0.481 (*0.999*) |
|  | Beta | 0.223 | -0.276 | 0.442 (*0.999*) |
|  | Lower Gamma | 0.196 | -0.311 | 0.369 (*0.999*) |
|  | Higher Gamma | 0.199 | -0.308 | 0.342 (*0.999*) |
| Working Memory | Theta | 0.230 | -0.245 | 0.412 (*0.999*) |
|  | Alpha | 0.231 | -0.184 | 0.452 (*0.999*) |
|  | Beta | 0.232 | -0.213 | 0.390 (*0.999*) |
|  | Lower Gamma | 0.195 | -0.299 | 0.448 (*0.999*) |
|  | Higher Gamma | 0.180 | -0.325 | 0.474 (*0.999*) |
| *Note.* Adj.: Adjusted. All beta values of predictor variables were not significant after FDR correction. Age and sex were included as covariates, as were all other covariates included in previous MVPA models examining absolute PSD. | | | | |

**Supplementary** **Table 5. SVR model results of predicting PPVT, and Maternal Education, without residualizing procedure.**

| Target | Predictors | Frequency bands | R^2^ score | *P*-value | Adj. *P*-value |
| --- | --- | --- | --- | --- | --- |
| PPVT | rsEEG + Age + Sex | Theta | 0.004 | 0.003 | 0.006 ** |
|  |  | Alpha | 0.083 | 0.001 | 0.002 ** |
|  |  | Beta | -0.074 | - | - |
|  |  | Low Gamma | -0.052 | - | - |
|  |  | High Gamma | -0.046 | - | - |
| Maternal Education | rsEEG + Age + Sex | Theta | 0.101 | 0.001 | 0.004 ** |
|  |  | Alpha | 0.154 | 0.001 | 0.004 ** |
|  |  | Beta | -0.038 | - | - |
|  |  | Low Gamma | 0.052 | 0.001 | 0.004 ** |
|  |  | High Gamma | 0.053 | 0.001 | 0.004 ** |
| *Note.* ** *P* < .01, * *P* <.05. rsEEG: resting state EEG. Adj.: Adjusted using Bonferroni correction. -: Not applicable. | | | | | |

**Supplementary** **Table 6. SVM model results of classifying Income, without residualizing procedure.**

| Target | Classifiers | Frequency bands | F1 score | *P*-value | Adj. *P*-value |
| --- | --- | --- | --- | --- | --- |
| Income | rsEEG + Age + Sex | Theta | 54.7 | 0.040 | 0.200 |
|  |  | Alpha | 21.2 | 0.832 | 1 |
|  |  | Beta | 49.2 | 0.257 | 1 |
|  |  | Low Gamma | 57.9 | 0.009 | 0.045 * |
|  |  | High Gamma | 67.2 | 0.001 | 0.005 ** |
| *Note.* ** *P* < .01, * *P* <.05. rsEEG: resting state EEG. Adj.: Adjusted using Bonferroni correction. -: Not applicable. | | | | | |

| 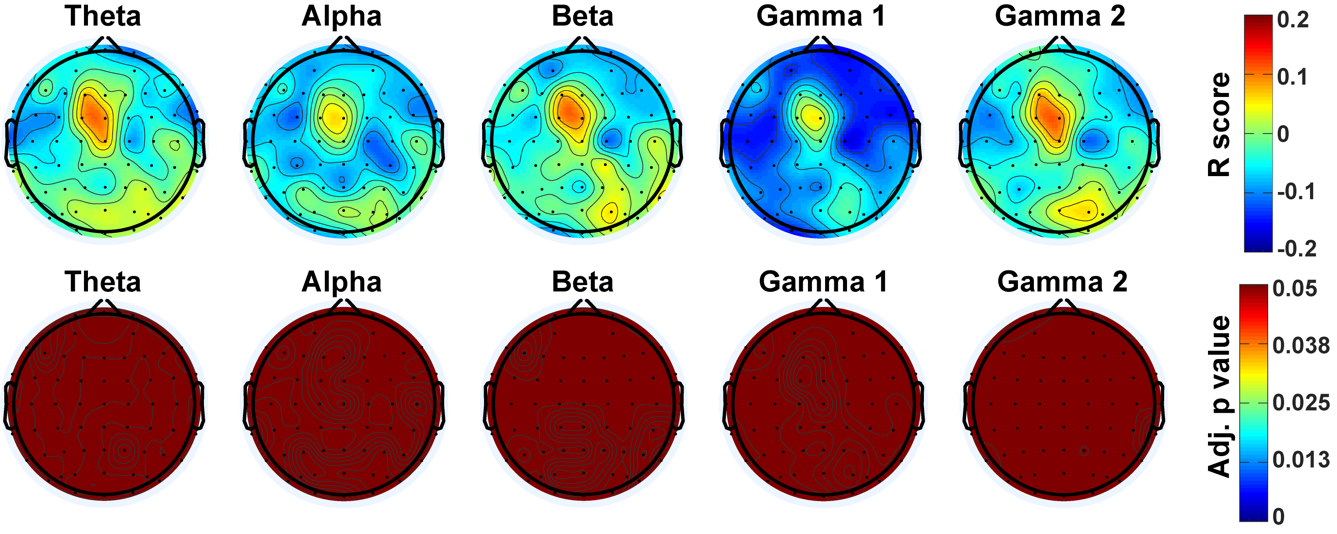  **Supplementary Figure 2. Topographies for the univariate correlation results of SES~PPVT and each channel of frequency bands.** The top row shows correlation coefficient (R score), and the bottom row shows FDR corrected *P-*value. |
| --- |
